# The Relation of the Advertiser to the Reader

**Published:** 1892-11

**Authors:** F. E. Daniel


For Daniel's Texas Medical Journal.

### THE RELATION OF THE ADVERTISER TO THE READER.

BY F. E. DANIEL, M. D.

A paper read by request before the Austin District Medical Society, at Austin, Texas, June 23, 1892.*

PEOPLE read for information; papers, journals and magazines are published to furnish that information. Some are general, and give the current news or information on all subjects, like the daily papers, but most publications are classified, and have a particular set or circle of readers. All trades, professions and callings have their appropriate jonrnals or exponents, and each class turn to their own particular journal for that which interests them or affects their business.

Since the earliest days of printing, advertisements have been resorted to as a means of communication between producer and consumer. Classification of advertisements has naturally followed, and to-day every trade journal and every paper devoted to the interests of any class of labor, calling or profession, has its own appropriate advertising department, through which information is conveyed to its readers of the advances, improvements and discoveries that take place in that particular line of industry.

The shoemaker naturally seeks in his *Leather Trade Journal* information concerning the implements, tools or appliances needed by him, and all improvements or advances made in his art. The stockraiser finds in journals devoted to that branch of industry not only all the news, advances and discoveries in connection with stock-raising, but he turns to its advertising depart-

*This paper is published in Dr. A. L. Hummel's book on Medical Journal Advertising.

ment to find offered any labor-saving implement or other thing needed in his business. He would never think of looking for it in the *Leather and Finding Journal*, or in the *Sanitary Journal*, any more than the housebuilder would expect to find the lumber market or building hardware in the *Churchman's Monitor*. The minister has his "church paper" with its advertising department, in which he finds offered such commodities as are needed by him, or will conduce to his comfort and conveniences; and so the number is limitless, and the divisions and subdivisions almost without end. Then there are literary magazines and papers read more or less by all classes. Their advertising department is a kind of *omnum gatherum;* they shoot with buck-shot, so to speak, hunt no particular game, but bring down anything in the range of their fire. In them you will find advertised toilet articles and saddlebags, bicycles and buggies, backgammon-boards and organs, pianos and land schemes, laces and looking-glasses, lounges and plants, chickens and typewriters, soothing syrup and shotguns, soap and sacred music, fountain pens and corsets, $3 pants and emulsion of cod liver oil. But in the main the latest and most approved tools, commodities and conveniences will be found in the advertising pages of that publication whose literature is intended for the class of readers who need these particular things.

The medical man should not be made an exception, nor should objection be made to the addition of an advertising department to his journal, wherein may be brought to his notice, not only any advance in the art of pharmacy, but any commodity he may need in his business or for his comfort or convenience.

When some eminent clinician like Da Costa or Roosa, Trousseau or Pepper, finds a certain combination of known drugs useful in certain proportion or combination, a paper, perhaps, is read on the subject at some medical society meeting. Some manufacturer then makes up this combination in convenient form, and puts it on the market, calling attention in the advertising pages of the medical journals to what has been said about it; and those physicians who are impressed order and use it. Is not the physician thus learning something from the advertising department of his medical journal? Before the rank and file of the medical profession had heard of sulphonal, ergotole, salol, iodol or phenacetin, the manufacturing chemists had learned all about them, made them ready for use, and naturally offered them to the profession through the medium in which a rational doctor would expect to hear of them—the medical journals. How, when or where else could he expect to learn anything of them?

Should the physician need a speculum, hypodermic syringe or other surgical instrument, a new fashioned buggy, an account book or an office coat, where would he expect to find it advertised for sale? Surely not in the shoemaker's journal; and, should it be any pharmaceutical preparation or a new product of the chemical laboratory, he certainly would not look in the *Daily Statesman* to find it advertised. You all know that the greatest offense, in the eyes of the medical profession, that a manufacturing chemist can be charged with, is that he advertises his products to the people. He says he manufactures his goods for the profession, for our convenience in practice, to save us labor or to facilitate our business; and you complain if he "goes to the fense," or puts his advertisement in a daily paper; and yet, some of you complain if you find his announcements in your medical journal. What *will* you have? *Suum cuique.*

The relation between the manufacturing pharmacist and the practitioner of medicine, is peculiar. While that of supply and demand obtains between them as consumers and producers, as in all other instances where one makes what another needs, there is furthermore a relation or connection that does not exist between any other two classes. Omitting the mention of all other manufactured articles a doctor may use, such as clothing, buggies, surgical instruments and appliances; not considering such things as he or his family need in common with other people, such as life insurance, house furnishing, pianos, books, paintings or clocks,—all of which might appropriately be advertised in his medical journal,--and confining our remarks solely to pharmacal products, we will hold and endeavor to show that the advertisements of such are not only appropriate to medical journals, but that they properly belong there, and nowhere else. The doctor is badly enough discriminated against in other ways, without depriving him of the privilege and satisfaction of finding in his medical journal, along with other useful and needed information, the details and particulars of the latest pharmaceutical products or triumphs, and where they can be obtained. He must learn of newly discovered drugs by seeing them offered for sale in his medical journal.

Pharmacy is a branch of medicine, and is so recognized. Two years ago the American Medical Association created a section of pharmacy for the especial purpose of bringing to the knowledge of physicians the progress, advances and discoveries in that branch of medicine, and to encourage physicians to become better acquainted with pharmacy and other branches of medicine, especially therapeutics, looking finally to a unification of medicine in all its branches. A physician should have some knowledge of pharmacy, as well as of chemistry, and the line heretofore separating pharmacy from medicine as a distinct branch is being obliterated. The pharmacist is not only the physician's supply man, but he is, to a great extent, his ally and assistant; their interests are closely identified; the relation is more intimately interwoven than that between any other supply man and his customers, for the doctor's success often depends upon the pharmacist's skill and honesty, while the health and happiness of the patient depend upon that of the doctor. The ignorant and dishonorable pharmacist having the physician's confidence and support, may ruin him. What more natural than that the practicing physician, realizing this dependence, should be interested in the methods and products, as well as in the advances and improvements in pharmacy? And if he does not learn of them through the advertising department of his journal, when, where, or in what way would or could he ever acquire that knowledge? You say "in journals of pharmacy," or "in the Dispensatory." Few physicians take or have time to read journals other than medical or sanitary, and should it be urged that to keep up with pharmacy he must take the pharmaceutical journals, the same obligalion would attach as well to journals devoted to any other line in which he is more or less interested; for instance, the clothiers' journal for his clothing, the carriage-builders' journal for his buggy and harness, etc. As to the Dispensatory, a discovery or advance in therapeutics or materia medica is many years old before it finds a place in that work, revised only every ten years. In brief, to the ambitious and progressive physician the medical journal is what the daily paper is to the business man, and its advertising department corresponds to the market reports and prices current; they are indispensable.

To the large majority of the profession the advertisements in medical journals of new preparations, or new combinations of old drugs, or favorite prescription of some eminent practitioner, which Sharp & Dohme, Parke, Davis & Co., or other enterprising manufacturers put on the market ready for use in convenient and attractive form are very interesting reading—indeed, they are invaluable. It is not every doctor who can write a prescription for, say, the poisonous drugs, the dose of which is infinitesimal, and with confidence send it to the drug store to be filled. It takes time to fill it, and there is always danger of a mistake in putting up such drugs; and, in the country, remote from drugstores, hours may elapse before the patient gets his medicine and the benefit of your skill. To one so circumstanced what a convenience and real labor saver it is to have tablets or pills in the proper doses, or concentrated extracts ready to administer without delay, danger or inconvenience. Why, then, should the manufacturer of pharmacals not advertise them to the physicians?

Why should the owners and manufacturers of proprietary articles, not advertise them in medical journals? You quote the Code "*to prescribe or recommend a secret nostrum is derogatory to professional character.*" The subject of secret nostrums is not under discussion, and no one contemplates recommending or prescribing them. But suppose that some publisher of a medical journal *should* advertise the most flagrant nostrum, does the Code prohibit your reading it, or does it provide a penalty for publishing it? You are no more obliged to read it—certainly no more obliged to buy the article and use it—than you are to purchase any other article you see advertised in any other journal. For instance we saw advertised in *The Statesman*, a "book for men" which we believed to be a vile quack swindle. The publishers of *The Statesman* do not necessarily recommend you to buy the book, and you are under no kind of obligation to do so. We do not argue that two wrongs make a right, but how often do we see quack advertisements, even immoral ones, in the daily papers, and in the religious press? Then why should you censure the publisher of a medical journal for publishing the advertisement of a "proprietary" article, perhaps of great value, like antipyrin, whose only objection is that it is covered by a trade mark?. Few proprietary articles, the ingredients of which are not known, are advertised in the better class of medical journals. Usually the only secret about them is the process of manufacture, and we hold that a knowledge of that alone (which is the proprietor's property) is not essential, if the ingredients are published, and the assurance is given that the dose is not disproportioned. We need no more care to know how it is made than to be told of the process of making the bread we ate for breakfast. Is the peach less sweet, or the diamond less brilliant because nature has not revealed the secrets of her laboratory?

So long as there is no one person or authority empowered to say which are, and which are not legitimate advertisements for a medical journal, each publisher must judge for himself. Because *The Journal of the American Medical Association* admits an advertisement to its pages of an article whose composition is unknown, that is no reason why other publishers should or should not do the same. Who shall be the censor? Aye, who?

Only recently the remarkable spectacle was witnessed of leading physicians—unimpeachable disciples of the Code—purchasing at enormous prices and introducing into the system of unsuspecting patients an animal substance—Koch's lymph—which was found to be highly poisonous, even deadly in its effects, unless used with extreme caution, the very composition of which, and its mode of preparation were entirely unknown to them.

Who was the first to use antipyrin—a secret nostrum patented, and therefore, outlawed in France? All our leading brethren in the United States have used it, and, if this be permitted, what blame can attach to a publisher who will take an advertisement offering these and similar preparations for sale?

When it is considered that medicine formerly was altogether, and is now largely empirical; that somebody has to first try a drug or chemical, I think it is rather "straining at a gnat" to say a physician shall not use proprietary articles the ingredients of which are known to the profession and known to be valuable, and which preparations, moreover, have been found to possess curative properties not to be secured from the use of either of the ingredients singly? We believe that ethical scruples should not prevent our using a trade-mark preparation which experience has shown will relieve certain conditions, for the ends and objects of the practice of medicine are to restore our patients to health. What the patient most cares for is that restoration; or, as the celebrated therapeutist and clinician, Fothergill, expressed it, "When a man is sick what he wants is to get well; the means is to him a matter of comparative indifference."

The objection has been raised that the manufacturing pharmacist dictates to the physician what he shall use, by offering a compound or combination ready for use. Such can hardly be called a rational objection. We cannot see it in that light. The maker of any of the articles usually advertised recommends them to your favorable consideration and asks you to give them a trial—perhaps furnishes you a sample for that purpose. If you have any scruples about the propriety of doing so, don't do it; but where is the objection to even a physician-publisher admitting the advertisement to his pages? Would a man buy counterfeit money if he saw it advertised? Would you stop your church paper, because it advertises quack medicine and quack doctors, with a portrait of that "old missionary to India whose sands of life had nearly run out," when he discovered a sure cure for consumption? Do you stop your *Galveston News* or *Louisville Courier-Journal*, because Drs. Betts & Betts advertise in them consultation free to the afflicted, and call attention to their numerous diplomas, which they say hang on their office walls; or because they advertise old Dr. Whittier who displays a wood cut of "Before Taking" and "After Taking" and guarantees to restore lost manhood? Or do you stop *The Statesman* because it advertises "a book for men only" wherein is offered free "a simple and safe device," whereby the sexual organs of the male may be brought to the highest state of functual activity and vigor, and those "shrunken from early indiscretions or stunted in their growth" maybe made to grow to full regular size and thus ensure the "Triumph of Love—a fruitful and happy marriage??" Do you place *The Evening News* under ban of proscription, because it offers "Tansy Wafers" to your wives, daughters and sisters? No; notwithstanding these highly disgusting and immoral advertisements, these papers—like Tennyson's brook—go on forever; are read in the family circle and allowed to poison the minds of your sons and daughters. But if your medical journal, be it ever so ethical, be it ever so meritorious in all else, should print the advertisement of some proprietary remedy, the howl goes up that it is "unethical."

As a final defense of the practice of advertising in medical journals we will mention that even *The Journal of the American Medical Association*, which has the backing of that great association and which receives five dollars annually from each member is compelled to admit that without the revenue from its advertisements it could not exist. The experience of the great majority of medical publications is of the same tenor; that is, they could not live a year if they depended solely upon their subscriptions for support; so by cutting off this revenue you would sound the death knell of the weaker vessels, and would virtually create a grasping, exacting monopoly of the few journals that survived. Is this desirable? True, there are too many; but would the fittest survive?

